# Regulation of histones in thromboinflammation

**DOI:** 10.3389/fimmu.2026.1791619

**Published:** 2026-04-01

**Authors:** Naila Mohiuddin, Yesha Shah, Saravanan Subramaniam

**Affiliations:** 1 Department of Pharmaceutical Sciences, Massachusetts College of Pharmacy and Health Sciences, Boston, MA, United States; 2 Renal Section, Department of Medicine, Chobanian & Avedisian School of Medicine, Boston University, Boston, MA, United States

**Keywords:** DAMPs, histone, NETs, PTMs, thromboinflammation

## Abstract

Extracellular histones, once regarded solely as nuclear structural proteins, are now recognized as potent mediators of thrombo-inflammation which is the pathological interface of coagulation and immunity. Released during necrosis, apoptosis, and neutrophil extracellular trap (NET) formation, histones act as damage-associated molecular patterns (DAMPs), engaging receptors such as Toll-like receptors (TLR2, TLR4, TLR9) to trigger endothelial dysfunction, platelet activation, and cytokine release. Post-translational modifications (PTMs), including citrullination, acetylation, and methylation, further modulate histone immunogenicity, cytotoxicity, and procoagulant potential. These mechanisms amplify thrombin generation, impair anticoagulant pathways, and promote vascular permeability, positioning histones as central drivers of immunothrombosis in sepsis, stroke, ARDS, COVID-19, and autoimmune disorders. Circulating histones and nucleosomes are emerging as biomarkers for disease severity and prognosis. Therapeutic strategies targeting histones, such as neutralizing antibodies, heparin derivatives, PAD inhibitors, and activated protein C, show promise in mitigating histone-driven pathology. This review highlights mechanistic insights into histone biology and explores translational opportunities for targeted interventions at the intersection of inflammation and thrombosis.

## 1 Introduction

Thrombo-inflammation is a complicated body response where inflammation leads to a higher risk of blood clots, which then makes the inflammation worse (1). Various cellular and molecular mediators that bridge the gap between hemostasis and immune activation emphasize the bidirectional complex interactions (2). Cells involved in thrombo-inflammation include platelets that are rapidly activated at sites of vascular injury or inflammation, neutrophils releasing a variety of pro-inflammatory mediators and forming neutrophil extracellular traps (NETs), monocytes transforming into macrophages and contributing to both inflammation and coagulation, and endothelial cells (ECs) whose dysfunction is a hallmark of thromboinflammatory states (3). These cells communicate and amplify responses through molecular mediators, including cytokines, chemokines, adhesion molecules, and components of coagulation cascade. Although the concept of immunothrombosis has a physiological role in coagulation system in innate immunity, when this system becomes dysregulated, it transitions into pathological thrombo-inflammation characterized by excessive and uncontrolled activation of coagulation, often within the microvasculature, leading to endothelial injury, impaired blood flow and ultimately, organ dysfunction and failure (1). Understanding the triggers and drivers of this transition is crucial for developing effective therapies. Thrombo-inflammation influences a wide array of clinical conditions, ranging from acute cardiovascular events like stroke, myocardial infarction, and deep vein thrombosis to infectious diseases such as COVID-19 (1, 4–6). Furthermore, it plays a critical role in the pathogenesis of autoimmune disorders, including systemic lupus erythematosus (SLE) and antiphospholipid syndrome (APS) (7–10). Collectively, these interactions result in vascular complications and thrombotic events that contribute significantly to morbidity and mortality in affected individuals (1, 4–10). The recognition of histones as key mediators is currently under active investigation due to its profound mechanistic and therapeutic implications in the context of thrombo-inflammation.

This narrative review provides a comprehensive and up-to-date examination of these complex mechanisms, highlighting the pivotal role of histones as central regulators and effectors. A structured literature search was conducted in PubMed, Scopus, and Web of Science, covering publications from January 1990 to January 2026, and including only studies published in English. Keywords and their combinations included Histones, post-translational modifications (PTMs), Inflammation AND Histone, Histones AND Diseases, Neutrophil extracellular traps (NETs), Damage-associated molecular patterns (DAMPs), Toll-like receptors (TLRs), Citrullination, Acetylation, Methylation, Immunothrombosis, Sepsis, Stroke, (Acute Respiratory Distress Syndrome) ARDS, (Coronavirus disease 2019) COVID-19, and Autoimmune disorders. Titles and abstracts were screened for relevance, and full texts were reviewed according to inclusion criteria: studies addressing histones in thrombo-inflammatory processes and post-translational modifications relevant to inflammation or coagulation, different diseases, and cell type-specific response. Excluded were non-English articles, conference abstracts without full text, and studies not directly related to histone-mediated thrombo-inflammation. Data on study objectives, experimental models, key findings, and conclusions were extracted and synthesized narratively to provide mechanistic insights, highlight translational relevance, and identify knowledge gaps in the field.

## 2 Histone biology

### 2.1 Histones structure, classification, and release mechanisms

**Figure 1:**
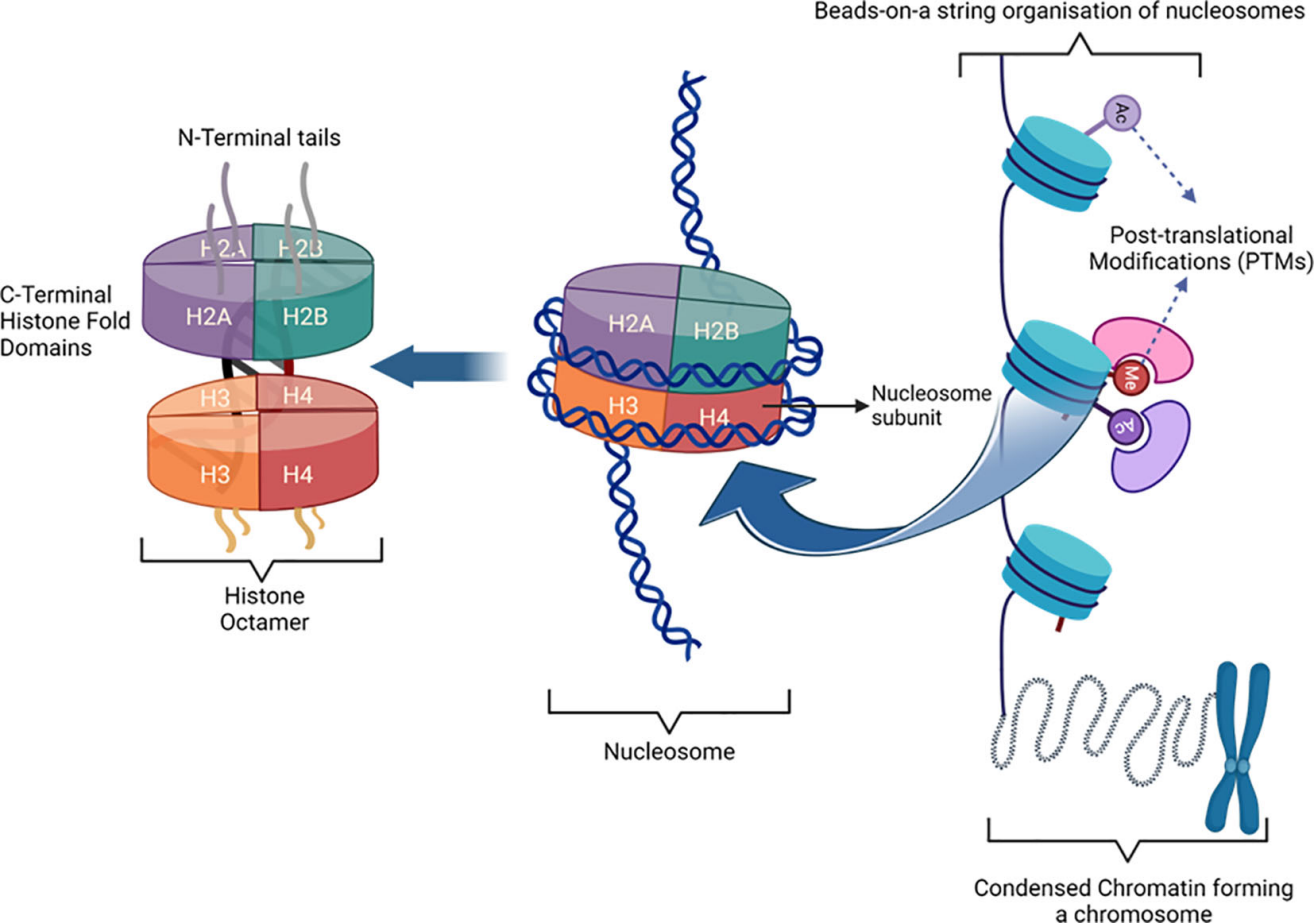
Histone composition and chromatin structure. The histone core consists of a (H3-H4)^2^ tetramer and two H2A-H2B dimers, forming an octamer around which 147 base pairs of DNA wrap twice, creating a nucleosome which is the basic unit of chromatin. Interactions with linker histone H1 and additional DNA segments contribute to higher-order chromatin structure. Around 75% of the histone mass comprises “histone fold” domains that support DNA wrapping and are highly conserved across species. The remaining 25–30% consists of flexible “tail” domains, which extend from the nucleosome surface and serve as sites for post-translational modifications (PTMs), crucial for regulating chromatin dynamics and gene expression.

Histones are basic, positively charged proteins composed of amino acid residues, such as arginine and lysine, and form nucleosomes, where DNA wraps around the histone core, consisting of two copies each of the histones H2A, H2B, H3, and H4 (11). Histones are classified based on their structural (linker vs. core) and chemical (lysine and arginine enrichment) composition (**Table 1**). Linker DNA connects nucleosomes and further compacts them into higher-order chromatin structures to facilitate DNA packing (**Figure 1**).

**Table 1:** Classification of histone types: H2A, H2B, H3, H4 are often referred to as the core histones because they form the nucleosome core around which DNA is wrapped to form chromatin while H1 mediates interactions between nucleosomes and is called the Linker histone protein.

Histone type	Structural classification	Chemical classification	Composition	Role
H1/H5	Linker Histone	Rich in both lysine and arginine residues	Large size	Higher-order chromatin structure and stabilization
H2A	Core Histones (Canonical forms)	Relatively rich in Arginine Residues	C-terminal and flexible N-terminal tail	Nucleosome stability, gene regulation, DNA repair
H2B			Long N-terminal tail and C-terminal domain	
H3		Rich in Lysine residues	Highly conserved histone fold domain with modifiable tail	
H4			Histone fold domain with modifiable tail	

H5 is a homologue of H1 found in different species.

Chromatin dynamism, a crucial trait for controlling nuclear activities such as transcription and replication, depends on DNA accessibility. The control of chromatin activity primarily relies on post-translational modifications (PTMs) of histones, involving enzymatic alterations to specific histone residues, particularly on the N-terminal tail. The presence of lysine and arginine residues facilitates various significant PTMs, including methylation, citrullination, acetylation, ubiquitination, phosphorylation, SUMOylation, glycosylation, and ADP-ribosylation, which are essential for epigenetic regulation, cellular development, and differentiation (12).

Histones are highly conserved proteins that play crucial roles in various biological functions. As nuclear proteins, histones facilitate the packaging of DNA into nucleosomes by acting as a scaffold for the negatively charged DNA, aiding in the processes of transcription and translation via their N-terminal tails (11). In response to cellular injury or signaling processes, extracellular histones are passively released during necrosis or apoptosis when the plasma membrane ruptures, which is associated with impaired phagocytosis (13). In addition, histones play a crucial role in innate immunity, being actively released from immune cells. Immune cells such as neutrophils and macrophages possess antimicrobial and pathogen recognition capabilities (14, 15).

Histones have been identified in the bloodstream of both healthy individuals (0.79–2.30 mg/L) and those suffering from conditions such as cancers, autoimmune disorders, inflammation, stroke, and sepsis (up to 230 mg/L) (16–18). Histones are released into the bloodstream during processes such as phagocytes undergoing NETosis to form extracellular traps (16, 17, 19). Damage-Associated Molecular Patterns (DAMPs) are molecules released by activated, damaged, or necrotic cells after injury, serving as signals of damage. Histones represent prime examples of DAMPs discharged from injured tissues into the bloodstream in significant amounts following severe trauma (20). Once in extracellular space, Histones can enter the bloodstream and act on distant cells to trigger thromboinflammatory responses. These extracellular histones (eHists) are released from cells during various pathological conditions, including extensive cell death (necrosis, apoptosis), cellular activation (e.g., NETosis by neutrophils), or severe tissue injury. eHists are now understood to function as critical alarmins that actively participate in intercellular communication and modulate host responses, particularly in the context of inflammation and coagulation (21).

This duality transitioning from essential nuclear components to potent extracellular mediators, places histones at a unique intersection of cellular homeostasis and pathology. Their release often signifies danger, linking cellular distress directly to systemic inflammatory and coagulation responses, which is central to their involvement in thrombo-inflammation.

### 2.2 Histones are a causal link to disease and are not merely biomarkers

Circulating histone levels are potential predictors of therapeutic response. In breast and lung cancer, patients with lower levels of circulating histones responded better to chemotherapy than patients with higher circulating histones levels (18). Following chemo or radiation therapy, decreased circulating histone levels were associated with disease regression, while elevated levels marked disease progression across several cancers (22). Neutralizing histones via antibodies, activated protein C, recombinant thrombomodulin, and heparin, have been indicated dampening the fatal endotoxemia, sepsis, ischemia/reperfusion injury, trauma, pancreatitis, peritonitis, stroke, coagulation issues, and thrombosis in murine models (23). These findings demonstrated that extracellular histones have a causal link to disease and are not merely biomarkers. Various pathways contribute to the toxicity associated with histones in thrombo-inflammation, including the activation of TLRs, elevation of membrane permeability, enhancement of transepithelial conductance, induction of calcium influx across diverse cell types, thereby promoting cytokine synthesis and release (including tumor necrosis factor-α, IL-6, and IL-10), and inhibition of macrophage phagocytosis (24–26). Nevertheless, there is still an incomplete understanding regarding potential variances in the cytotoxic effects of specific histone subtypes, as well as disparities in establishing the exact mechanism of how these integral components supplement but also augment inflammation and the immune system.

### 2.3 Sources and triggers for extracellular histone release

**Figure 2:**
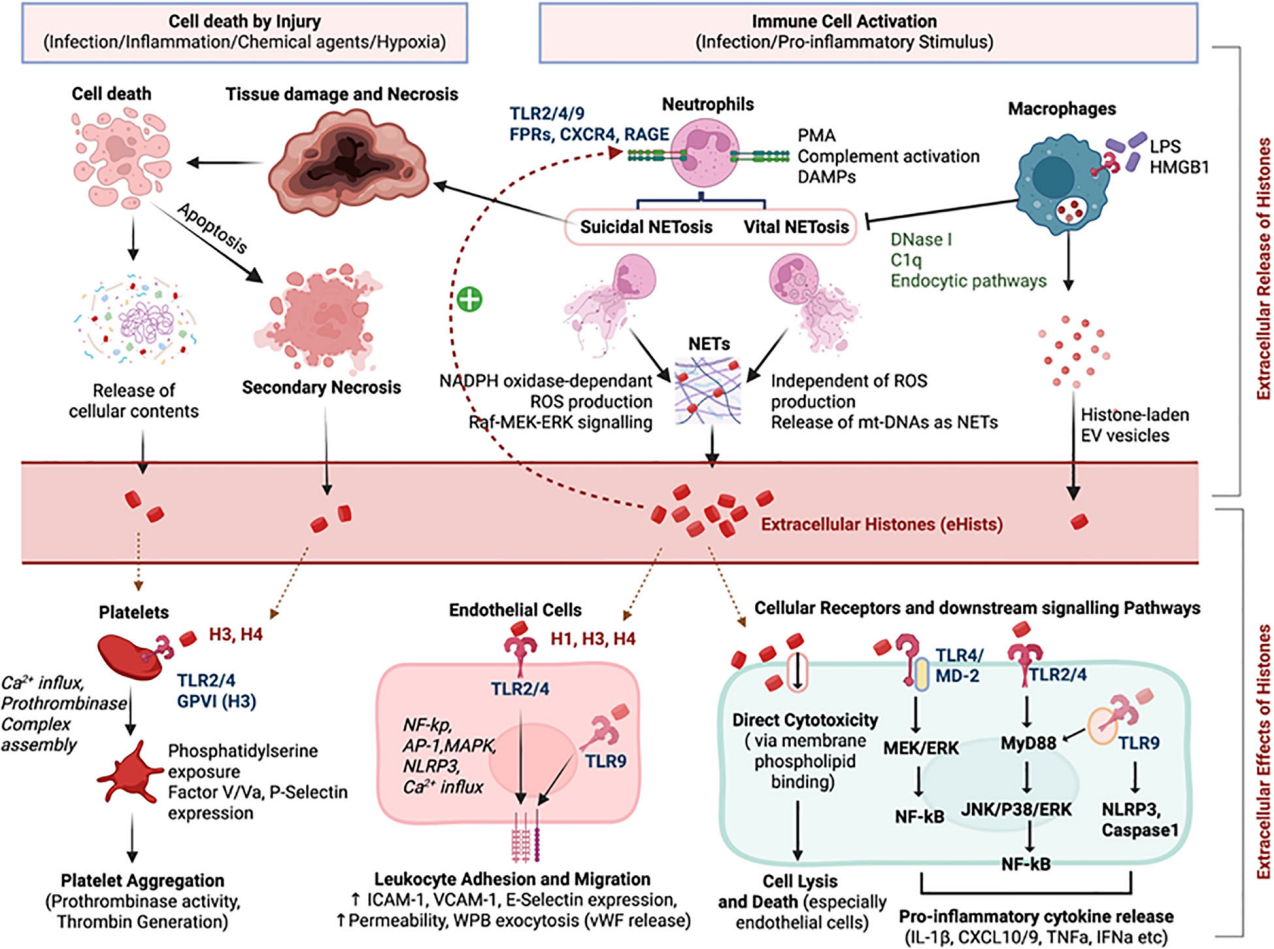
Mechanisms of extracellular histone release and downstream thromboinflammatory signaling. Extracellular histones, released via neutrophil extracellular trap (NET) formation (through ROS-dependent “suicidal” or ROS-independent “vital” pathways) and cell necrosis, act as damage-associated molecular patterns (DAMPs) to drive thromboinflammation. (Left) Upon release, histones (specifically H3, H4) activate platelets through TLR2/4 and GPVI binding, triggering Ca²^+^ influx, phosphatidylserine exposure, and prothrombinase complex assembly, culminating in thrombin generation and aggregation. (Right) On endothelial cells, histones stimulate TLR2/4/9 and NLRP3 inflammasome signaling via MyD88 and NF-κB/MAPK pathways. This induces a pro-inflammatory phenotype characterized by cytokine release (IL-1β, TNF-α), upregulation of adhesion molecules (ICAM-1, VCAM-1), and Weibel-Palade body exocytosis (vWF release), while high concentrations cause direct cytotoxicity and cell lysis. Abbreviations: DAMPs, damage-associated molecular patterns; GPVI, glycoprotein VI; NETs, neutrophil extracellular traps; NLRP3, NOD-like receptor family pyrin domain containing 3; ROS, reactive oxygen species; TLR, Toll-like receptor; vWF, von Willebrand factor.

Uncontrolled cell death, such as necrosis, resulting from severe injury, infections, trauma, ischemia-reperfusion injury, sterile inflammatory conditions, autoimmune diseases, and certain therapeutic interventions, leads to the rupture of plasma membranes and the passive release of intracellular contents, including histones, into the surrounding environment (27). Apoptosis, or programmed cell death, is generally considered immunologically silent due to the formation of apoptotic bodies that are efficiently cleared by phagocytes. However, if phagocytic clearance is impaired or overwhelmed, late apoptotic cells can undergo secondary necrosis, also leading to histone release (13, 28). NETosis, a specialized process primarily executed by neutrophils, although other immune cells like mast cells and macrophages can also release extracellular traps. NETs are web-like structures composed of decondensed chromatin (DNA and histones) decorated with antimicrobial proteins and enzymes. Histones are the major protein components of NETs. (**Figure 2**). Emerging evidence indicates that macrophages, when stimulated with LPS, can actively release histones into the extracellular milieu (14). This release occurs both in soluble form and, significantly, in association with extracellular vesicles (EVs), including micro-vesicles and exosomes (14, 29). These EV-associated histones, of nuclear origin, are frequently located on the outer surface of the vesicles, positioned to interact with surrounding bystander cells.

Collectively, these multifaceted mechanisms of passive release during necrosis or apoptosis, active secretion via NETosis, and possible transport by EVs demonstrate extracellular histones are not just incidental byproducts of cell death. Rather, they might be actively positioned signaling molecules with strong immunomodulatory potentials.

## 3 Post-translational modifications of histones and inflammation

In the 1960s, *Vincent Allfrey* et al. discovered that histone post-translational modifications (PTMs) primarily regulated chromatin activity (30). Among these, histone acetylation and methylation play pivotal roles in modulating chromatin structure and gene expression through RNA synthesis (31). Recent research has expanded the classical understanding of the “histone code,” showing that PTMs not only regulate histone function within the nucleus but also significantly influence the extracellular activity of histones during thrombo-inflammation (32, 33). These modifications act as a secondary regulatory layer that defines the specific inflammatory and prothrombotic potential of eHists (34). These modifications involve enzymatic alterations to specific histone residues, most notably concentrated on the N-terminal tails. Such covalent changes modulate the function of chromatin fibers, with distinct modifications leading to diverse functional outcomes (35).

Histone modifications influence gene expression by modulating DNA accessibility to the transcriptional machinery, either promoting or repressing transcription. For instance, histone acetylation is generally associated with transcriptional activation, while histone methylation can result in either activation or repression, depending on the specific residue modified and the degree of methylation. Additionally, these modifications often act as docking sites for proteins that regulate chromatin structure and gene expression, thereby impacting a wide range of cellular processes such as development, differentiation, and disease. Among the various PTMs, histone acetylation and methylation are the most extensively studied and understood (36).

Histone acetylation happens when the enzymatic addition of acetyl groups (CH_3_CO) to lysine residues on histone tails. Histone acetyltransferases (HATs) add, and histone deacetylases (HDACs) remove these acetyl groups. The acetylation process neutralizes the positive charge on histones, reducing their affinity for negatively charged DNA. This results in a more relaxed and open chromatin structure, making it accessible to transcriptional machinery and facilitating gene expression. Histone acetylation also plays a pivotal role in inflammatory responses. Acetylation of histones H3 and H4 at promoters of pro-inflammatory genes (e.g., TNF-α, IL-6, IL-1β) enhanced the recruitment of NF-κB, a key transcription factor in inflammation (37). Additionally, phosphorylation of histone H3 at serine 10 (H3S10ph), mediated by mitogen- and stress-activated kinases (e.g., MSK1/2), is associated with immediate-early gene activation during stress and inflammation (38, 39). Extracellularly, histone acetylation status can influence histone release. For instance, histone acetylation can promote NETosis under baseline conditions and when induced by both NADPH oxidase (NOX)- dependent and -independent agonists (40). However, very high levels of histone acetylation, often achieved through the use of HDAC inhibitors can paradoxically suppress NETosis and promoted a switch from NETotic to apoptotic cell death in neutrophils suggesting a complex role for acetylation in determining not only gene expression but also the method of DAMP release and the mode of cell death (40).

In contrast, histone methylation involves histone methyltransferases (HMTs), which target specific lysine or arginine residues on histones H3 and H4, adding methyl groups (CH_3_) on them which are reversed by Histone demethylases (HDMs). This modification is considered a stable epigenetic mark, making histones more basic and hydrophobic, thereby increasing their affinity for DNA. Histone methylation can have varying effects on transcription depending on the specific residues methylated and the number of methyl groups (mono-, di-, or tri-methylation) (41).

The first histone methyltransferase (HMT) and histone demethylase were identified in 2000 and 2004, respectively (42, 43). These enzymes have opposing functions, highlighting the dynamic nature of histone methylation. Methylation of H3K4 (the fourth lysine on histone H3) is associated with gene activation, whereas methylation of H3K9 (the ninth lysine on histone H3) is linked to gene repression (44, 45).

Histone methyltransferases are classified into protein lysine methyltransferases (PKMTs) and protein arginine methyltransferases (PRMTs). Enzymes such as Jmjd6, SETD2, EZH2, G9a, and PRDM6 play critical roles in vascular development and maturity (46–48). Notably, H3K4me3, a methylation mark at the fourth lysine residue of histone H3, is commonly found at promoters of pro-inflammatory genes, enhancing their expression (49). This modification has been observed in the synovial tissues of rheumatoid arthritis patients, correlating with inflammatory cytokine expression (50). Conversely, H3K27me3, a methylation mark at the twenty-seventh lysine residue of histone H3, is associated with gene repression and can dynamically regulate inflammatory gene expression (51).

Citrullination, also known as deamination, is catalyzed by Peptidyl Arginine Deiminase (PAD) enzymes, particularly PAD4 in neutrophils. This modification converts arginine residues on histones into citrulline, neutralizing their positive charge and thereby weakening histone-DNA interactions. This relaxation of chromatin structure is a prerequisite for NET formation. Among citrullinated histones, citrullinated histone H3 (CitH3) is widely recognized as a hallmark of NETosis and is frequently employed as a biomarker in both experimental and clinical settings (52–54). Extracellular CitH3 is a potent pro-inflammatory and pro-thrombotic mediator that activates ECs and platelets, often via TLR4, and its circulating levels correlate with disease severity in sepsis, deep vein thrombosis, and Disseminated Intravascular Coagulation (DIC) (55). Beyond acetylation, methylation, and citrullination, histone phosphorylation and lactylation also regulate inflammatory and vascular responses. Notably, H3S10 phosphorylation promotes chromatin remodeling and immediate-early gene activation during cellular stress, enhancing pro-inflammatory transcription via MAPK and NF-κB pathways (38, 39). Histone phosphorylation influences both nuclear gene expression and the inflammatory potential of extracellular histones. Similarly, histone lactylation (e.g., H3K9la) links cellular metabolism to epigenetic regulation, as evidenced by VEGF-induced glycolysis in ECs, which promoted cell proliferation and angiogenesis. This metabolic priming serves as a critical bridge between anaerobic states and inflammatory neovascularization (56).

Distinct histone PTM profiles regulate cytotoxicity, receptor engagement, and procoagulant activity. These dynamic modifications are controlled by PTM “writers” enzymes (such as HATs, HMTs, PADs, and kinases) that respond to cellular signaling and metabolic cues, enabling context-specific inflammatory responses. Crosstalk between PTMs, such as H3S10ph with H3K14ac, enhances chromatin remodeling and transcription, exacerbating thrombo-inflammation, while epigenetic memory can sustain pro-inflammatory gene expression and promote chronic inflammation (48, 57, 58).

Histone modifications have clinical implications. Anti-histone antibodies serve as important markers for distinguishing different forms of lupus, such as idiopathic and drug-induced lupus (59). Drug-induced histone modifications may also trigger autoimmunity, potentially involving neutrophil mechanisms (60–62). Histone acetylation has been implicated in stiffness-induced gene expression in liver sinusoidal ECs, promoting inflammation, edema, and increased portal venous pressure (39). Acetylation of histone H4K16 has been linked to EC senescence through telomere shortening (63).

Histone PTMs profoundly alter receptor affinity, cytotoxicity, and immunogenicity. Notably, CitH3 is a potent DAMP and TLR4 agonist that amplifies pro-inflammatory cytokine production through charge neutralization and altered receptor interactions. PTMs also influenced histone cytotoxicity, as citrullinated NET-derived histones were less susceptible to neutralization than unmodified forms, with important implications for therapeutic efficacy (64, 65). CitH3 correlated with NET-driven thrombosis, by promoting platelet activation and fibrin formation (66). Emerging PTMs such as histone lactylation link cellular metabolism to chromatin dynamics, suggesting that metabolic states like enhanced glycolysis pre-shape histone PTM profiles before release (67).

These insights into histone modifications underscore their pivotal role in transcriptional regulation, inflammation, and disease, providing potential therapeutic targets for epigenetic intervention.

## 4 Histones as DAMPs

Once histones are in extracellular space, they transition from chromatin structural proteins to potent DAMPs, initiating and perpetuating inflammatory responses even in the absence of infection. Extracellular histones exert their DAMPs effect through several mechanisms (**Figure 2****).** Due to their highly positive charge (rich in lysine and arginine residues), histones can interact with negatively charged components of cell membranes, such as phospholipids (68). This interaction disrupts membrane integrity, resulting in increased permeability, uncontrolled calcium influx, and eventually lead to cell lysis and death. ECs are particularly vulnerable to this direct cytotoxicity (17). Histone binding to anionic membrane phospholipids occurs in stoichiometric ratios with high concentrations disrupting the lipid bilayer of the cells (69). This direct cytotoxicity is dose dependent- while high doses of histones (e.g., 50ug/mL or higher) cause severe endothelial damage and reduced cell viability through these direct membrane effects, lower doses induce autophagy and apoptosis in ECs via mammalian target of rapamycin (mTOR) signaling (70). This direct membrane disruption appears to be a primary mechanism of histones, as studies suggest that histones cytotoxicity may not strictly require interaction with cell surface glycosaminoglycans (GAGs) like heparin sulfate (71). Extracellular histones can also act as chemoattractants, drawing neutrophils and monocytes to sites of injury or inflammation (72). In addition, the process led to nearby cells to release secondary chemokines, further amplifying immune cell recruitment and disseminating the inflammatory response.

Beyond direct membrane disruption, eHists elicit specific cellular responses by engaging various pattern recognition receptors (PRRs) expressed on both immune and non-immune cells (73). TLRs represent a major class of PRRs that recognize and respond to eHists. TLR2 and TLR4 sense eHists on ECs, platelets, neutrophils, and macrophages; research demonstrated that eHists act as potent inducers of sterile inflammatory injury through the activation of TLR2 and TLR4, expressed on immune and vascular cells (73) (**Figure 2**). Using two mouse models Concanavalin A-induced sterile inflammation and acetaminophen-induced liver injury the study showed that eHists released during tissue damage contributed directly to leukocyte recruitment, cytokine storm, and organ failure (74). Notably, the interaction between eHists and these receptors is often enhanced when histones are complexed with DNA, as seen in nucleosomes released during NETosis or cell death suggesting that the structural context in which histones are released (free histones or as part of nucleosomes) is a critical determinant of their immunomodulatory activity (75). Nucleosomes may act as more potent DAMPs by providing a scaffold for TLR clustering or co-receptor engagement.

Additionally, TLR9, typically known for recognizing unmethylated CpG-rich DNA within endosomes, has also been implicated in histone-mediated signaling. In particular, histone interactions with TLR9 contributed to neutrophil activation and inflammasome engagement in Kupffer cells (76, 77). Histones may facilitate DNA delivery to TLR9 containing endosomes or may engage the receptor through alternative mechanisms. They promote DNA curvature, increasing binding efficiency of TLR9 and downstream pro-inflammatory signaling particularly via DNA curvature-inducing proteins including high-mobility group box 1 (HMGB1) and histones H2A and H2B (78). The ability of histones to activate multiple TLRs underscores a robust and potentially redundant danger-sensing system, likely designed to ensure a swift immune response across various pathological contexts which is yet to be investigated in detail.

Following PRR engagement, downstream signaling cascades are initiated, often involving adaptor proteins such as MyD88 and leading to the activation of key transcription factors like NF-κB and AP-1 resulting in transcriptional upregulation and release of a broad range of pro-inflammatory cytokines (e.g., TNF-α, IL-1β, IL-6) and chemokines (e.g., IL-8, MCP-1, CXCL9, CXCL10) (73, 77, 79). Furthermore, eHists also promoted the activation of the NLRP3 inflammasome, a cytosolic multiprotein complex responsible for processing and maturing IL-1β and IL-18, thereby further amplifying the inflammatory response (77). While TLRs remain the primary focus in histone recognition, other receptors such as the Receptor for Advanced Glycation End products (RAGE) may also play a role, although evidence for direct histone binding to RAGE is currently less robust compared to TLR-mediated mechanisms (80).

While TLR2, TLR4, and TLR9 are recognized as principal sensors of extracellular histones, the impact of their physical state- free versus nucleosome-bound remains poorly defined. This distinction is mechanistically critical because DNA-associated histones preferentially engage endosomal pathways, whereas free histones interact directly with the plasma membrane and surface receptors. The dual capacity of histones to induce membrane permeabilization and activate redundant TLR pathways suggests that their bioactivity is governed by strict spatial and biochemical controls. While the TLR-MyD88 axis provides the primary inflammatory framework, signaling intensity and specificity are likely dictated by the biochemical state of the proteins. Beyond concentration, the structural landscape of the histone molecule-specifically its PTMs dictates receptor affinity and drives the diversity of observed sterile inflammatory responses.

## 5 Cellular mechanisms of histone-driven thrombo-inflammation

Extracellular histones orchestrate a complex, multicellular response that fuels the cycle of thrombo-inflammation. They do not act in isolation but rather engage in detrimental crosstalk with ECs, platelets, neutrophils, and macrophages, each interaction contributing uniquely to vascular dysfunction, hypercoagulability, and sustained inflammation. This coordinated cellular onslaught underscores the formidable role of histones as potent systemic mediators of immunothrombosis. The following subsections detail these cell-specific mechanisms, exploring how histones transform the vascular landscape from a quiescent state to a pro-thrombotic and pro-inflammatory environment.

### 5.1 Histones and ECs in vascular function

ECs form a semi-permeable barrier between blood and tissues, maintaining vascular integrity and modulating hemostasis, inflammation, and angiogenesis. ECs respond dynamically to mechanical and biochemical stimuli, releasing mediators that influence vascular tone and immune responses. Dysfunction of ECs is a hallmark of many vascular disorders (81–83). Extracellular histones are potent instigators of endothelial activation and injury, transforming the normally quiescent endothelial lining into a pro-inflammatory and procoagulant surface (84).

Histones induced a procoagulant phenotype in ECs by dose-dependently increasing tissue factor (TF) expression and activity while decreasing thrombomodulin (TM) levels and activity, thereby promoting thrombin generation through TLR2 and TLR4 signaling, with inhibitors of these receptors partially reversing histone-induced TF up-regulation (85). Notably, histones H3 and H4 were the most potent in causing endothelial damage, with significant procoagulant activity observed at concentrations as low as 50 μg/mL (86). In addition to their procoagulant effects, histones also stimulated cytosolic reactive oxygen species (ROS) production in ECs in a concentration-dependent manner, with ROS generation promoting inflammatory responses via COX/NOX pathways and TLR4. Inhibition of these pathways mitigates ROS production and reduces the activation of NF-kB, as well as the expression of adhesion molecules like VCAM1 and ICAM1 (87, 88) (**Figure 2**). *Ramasubramanian* et al. demonstrated that histones, again with H3 and H4 being particularly potent, increased endothelial permeability by disrupting adherens junctions (e.g., VE-cadherin) and reorganizing the actin cytoskeleton leading to vascular leakage, edema formation, and the extravasation of plasma proteins and fluid into tissues, exacerbating inflammation and tissue damage (88). At high concentrations, histones can induce EC apoptosis and autophagy, further contributing to barrier breakdown and endothelial denudation (89). Another important mechanism of histone-induced endothelial activation is the exocytosis of Weibel-Palade bodies (WPBs) (90). WPBs are storage granules within ECs that contain von Willebrand factor(vWF) and P-selectin, among other pro-inflammatory and pro-thrombotic mediators. Histone-induced WPB degranulation releases large vWF multimers, which are crucial for platelet adhesion and aggregation, especially under high shear stress, and P-selectin, which further promotes leukocyte recruitment (86).

Extracellular histones also trigger autophagy and apoptosis in ECs, with dose-dependent effects on cell viability (74, 89, 91). Molecules such as chondroitin sulfate have been suggested as potential therapeutic agents to neutralize histones and prevent the lethal thrombosis induced by their interactions with platelets, neutrophils, ECs, and the coagulation system (91). Moreover, histones have been implicated in modulating the immune response by stimulating the expression of inflammatory markers, such as Intercellular Adhesion Molecule-1 (ICAM-1), Monocyte Chemoattractant Protein 1 (MCP-1), and IL-6, and expanding specific regulatory T cell populations, such as FoxP3^hi^ (85). The potential role of G protein-coupled receptor 109A (GPR109A, also known as HCA2) on ECs in modulating these responses is an area of speculation. GPR109A is recognized for its anti-inflammatory effects, primarily mediated through immune cells (92). Intracellular VEGF signaling coupled metabolism to epigenetics by enhancing glycolysis and H3 lysine 9 lactylation (H3K9la), a modification essential for endothelial proliferation and tube formation. Consequently, targeting the H3K9la pathway either through metabolic inhibition or HDAC2 overexpression significantly impaired angiogenesis, offering a novel therapeutic strategy for pathological vascularization (93). These findings underscore the complex role of histones in EC dysfunction and thrombosis, highlighting potential therapeutic strategies to mitigate histone-induced vascular damage.

### 5.2 Histone-mediated platelet activation and its role in thrombo-inflammation

Platelets, anucleate cells primarily involved in hemostasis, play a crucial role in maintaining vascular integrity and preventing thrombosis under normal conditions (94, 95). Platelets function by releasing clotting factors, including vWF and factor XIII, and providing a scaffold for the activation of prothrombin (96). Platelet activation is tightly regulated by endothelial-derived antagonists such as CD39, prostacyclin, and nitric oxide, which prevent inappropriate platelet adhesion and aggregation, ensuring smooth blood flow (97). However, under pathological conditions such as sepsis, ischemia–reperfusion injury, and DIC, platelets sense pathogens and damage signals via PRRs, including TLRs and integrins, triggering shape changes that promote interactions with leukocytes, particularly neutrophils (98). This interaction facilitates neutrophil extravasation and activation, ultimately resulting in the formation of neutrophil extracellular traps (NETs), which are a major source of extracellular histones (99). Histones induced thrombocytopenia and platelet activation, which correlated with poor prognosis in patients diagnosed with sepsis (100). Elevated levels of platelet-associated histones were observed in septic patients, suggesting that activated platelets contribute to circulating histone levels (101). Importantly, histones bound to platelets via TLR2 and TLR4, increased platelet aggregation, expression of P-selectin (CD62P), phosphatidylserine, and factor V/Va, as well as enhanced prothrombinase activity (102–104).

Extracellular histones drive chronic fibrosis by triggering a platelet–macrophage feed-forward loop (105). In a model of pulmonary fibrosis, histone-activated platelets released TGF-β1, which stimulated macrophage IL-27 production, to drive fibroblast activation and matrix deposition, identifying a novel therapeutic target to disrupt fibrotic progression (105).

This platelet-neutrophil crosstalk establishes a reciprocal axis where histone-activated platelets trigger NETosis, thereby replenishing the extracellular histone pool and sustaining the thrombo-inflammatory cycle.

### 5.3 Histones-mediated activation of neutrophils in thrombo-inflammation

Neutrophils and dead cells are the primary source of extracellular histones, releasing them through the formation of NETs via a process known as NETosis. About 20-60% of neutrophils released NETs, primarily with the help of the enzyme Peptidyl Arginine Deiminase 4 (PAD4), which converted arginine to citrulline (106). Citrullination facilitates chromatin decondensation, by loosening histone-DNA interactions thereby enabling the extrusion of chromatin to form NETs. NETs provide a scaffold for antimicrobial peptides and enzymes that immobilize and kill pathogens (107). NET-derived products, including histones, DNA, and HMGB1 activated inflammasomes in macrophages, leading to the release of IL-1β and IL-18, cytokines known to further stimulate NET formation (108).

Based on differences in the stimuli that activate neutrophils and the signaling pathways involved, *Zhou* et al., classified NETosis into two categories: suicidal and survival NETosis. Suicidal NETosis, or conventional NETosis, occurs when histones are released through cell death resulted in the complete destruction of neutrophils within 2–20 hours of stimulation via NADPH-dependent ROS production (109, 110). In contrast, survival NETs involved the release of vesicular DNA within an hour of stimulation by *Staphylococcus aureus*, while retaining their phagocytic abilities (111). Suicidal NETosis was triggered by Fc receptors, TLRs, and complement receptors, leading to ROS generation, PAD4-mediated chromatin citrullination, membrane rupture, and NET release. In contrast, vital NET formation occurred via TLR2/4 signaling, activated PAD4 independently of ROS, and released NETs through vesicular exocytosis without cell lysis (112).

While histones effectively neutralize pathogens, they are cytotoxic to host cells, including neutrophils. Certain bacteria, such as secreting leucocidins like ionomycin, have evolved mechanisms to evade this defense (113). In addition to directly inducing cytotoxic effects by disrupting cell membranes through their positive charge, histones act as DAMPs, activating neutrophils through TLR2, TLR9, formyl peptide receptors (FPRs), CXCR4, and RAGE (71) (**Figure 2**). This activation triggers cytokine production, oxidative stress, and the release of NETs, creating a vicious cycle. *Nakazawa* et al. demonstrated that histones released during hypoxia-induced tubular epithelial cell necrosis primed neutrophils for NETosis, induced further tubular epithelial cell death (114). In its mouse model of ischemia-reperfusion injury, pretreatment with anti-histone IgG suppressed NET formation, reduced renal injury, and decreased the accumulation of TUNEL-positive cells in the lungs, liver, brain, and heart, along with NET accumulation in the lungs (114).

*Yousefi* et al. showed that neutrophils can release mitochondrial DNA to form NETs without dying, a process called vital NETosis. Primed by GM-CSF and activated via TLR4 or C5a receptors, this ROS-dependent mechanism produces mtDNA NETs independent of nuclear DNA and neutrophil death (115).

Neutrophil elastase (NE) and myeloperoxidase (MPO)are key mediators in facilitating chromatin decondensation during NETosis. Upon NETotic stimulation, NE translocated to the nucleus, where it cleaved core histones such as histone H4 promoting chromatin relaxation and nucleosomal dismantling (116). MPO, while classically known for its microbicidal oxidative functions, is also implicated in the NETotic process. Although its exact mechanistic role is context-dependent, MPO may facilitate NE activity or directly modify histones and DNA through oxidative mechanisms (116). Recent studies highlight gasdermin D as a key regulator of NETosis, mediating plasma membrane rupture during lytic NETosis and facilitating NE translocation to the nucleus, coordinating chromatin decondensation and extracellular histone release (117–119).

*Leung* et al. showed that heparin-induced thrombocytopenia (HIT) immune complexes, triggered ROS-dependent NETosis via FcγRIIa. Inhibiting the ROS generating NOX2, with DPI or GSK2795039 reduced NETosis and thrombosis in mice without affecting thrombocytopenia, highlighting ROS has a central role (87, 120). Neutrophils not only release extracellular histones via NETosis but can be further activated by them, creating a self-perpetuating cycle. Histone modifications, such as citrullination, acetylation, and methylation, regulate NETosis. Acetylation generally promotes NET formation, though high levels (e.g., via HDAC inhibitors) may suppress NETosis and induce apoptosis. Methylation changes on NET-associated histones may influence chromatin decondensation and NET stability (40, 121).

Understanding the precise triggers and regulatory mechanisms of different NETosis pathways is crucial for developing strategies to mitigate their detrimental effects while preserving their beneficial antimicrobial functions.

### 5.4 Histone mediated activation of monocytes and macrophages

Circulating monocytes serve as the primary innate immune sensors of extracellular histones within the vasculature. As potent DAMPs, eHists trigger the recruitment of these monocytes to sites of endothelial injury or inflammation (122).

Xu and colleagues established that extracellular histones mediate cellular toxicity and systemic inflammation in monocytes primarily through TLR2 and TLR4 (74). On monocytes, eHists binding to these receptors initiates the MyD88-dependent signaling cascade, leading to the nuclear translocation of NF-κB and the subsequent transcription of pro-inflammatory cytokines (IL-1β, IL-6, and TNF-α) (74) (**Figure 2**). Histone H4 functions as an early innate immune sentinel by directly interacting with the TLR4/myeloid differentiation factor 2 (MD-2) complex on human monocytes triggering rapid downstream signaling characterized by the mobilization of chemotactic chemokines CXCL9 and CXCL10, indicating a role for extracellular histones in amplifying leukocyte recruitment (75). Histones have also been demonstrated to shift monocytes to procoagulant phenotype. Upon TLR-2 and TLR-4 activation by histones, intracellular signaling via NF-κB and AP-1 pathways forces surface expression of TF on human blood monocytes (123). This enhances localized thrombin generation, effectively bypassing normal endothelial regulatory mechanisms and accelerating microvascular thrombosis (124). Additionally, the P-selectin expressed by histone-mediated-platelet activation binds to the pre-existing PSGL-1 on the monocytes, physically locking them together into a Monocyte-Platelet Aggregate (MPA) creating a localized positive feedback loop (125, 126). The physical binding amplifies superoxide anion production and supercharges the release of monocyte chemotactic protein-1 (MCP-1) and TF, accelerating clot formation (127). Extracellular histones are associated with clinically measurable monocyte alterations. *Daniela Ligi* et al. showed that recombinant histones increased Monocyte Distribution Width (MDW) in human whole blood, reflecting changes in monocyte size, granularity, and nuclear morphology similar to those seen in viral and classical sepsis. These findings support extracellular histones as upstream drivers of hyperinflammatory monocyte changes linked to multi-organ dysfunction (128, 129).

However, even as they undergo this structural dysregulation, the innate immune system simultaneously attempts to mount a protective defense. Recent clinical and experimental studies have identified Clusterin (CLU) as an endogenous counter-regulatory chaperone that neutralizes extracellular histone toxicity (130–132). In sepsis models and patient cohorts, CLU was shown to directly bind circulating histones and reduce histone-mediated cytotoxicity and inflammatory injury. Although monocyte CLU expression increases as a compensatory mechanism, this response is insufficient in severe disease. Following tissue infiltration, monocyte-derived macrophages further sustain thromboinflammation through cytokine production (133).

Histones whether free, NET-bound, or packaged within extracellular vesicles (EVs) activate macrophages via PRRs, particularly TLR4, triggering the release of cytokines such as TNF-α, IL-1β, and IL-6, and thereby perpetuating inflammation (73). Macrophages stimulated with LPS released histone-loaded EVs that activated naive macrophages in a paracrine fashion, further amplifying the inflammatory loop (134). NET-derived DNA, HMGB1, and histones activated the NLRP3 inflammasome in macrophages, driving the production of IL-1β and IL-18 to promote further NET formation (135). High mobility group box 1 (HMGB1) regulated the density of histones and nucleosomes that package DNA and is actively secreted by immune system cells, including monocytes, dendritic cells, natural killer cells and macrophages (136). *Toma* et al. showed that LPS stimulation of wild-type FLDMs decreased nuclear histone H3, which correlated with HMGB1 release, linking HMGB1 dynamics to histone levels in inflammation. Follow-up studies reported a time-dependent ~20% reduction in nuclear H3, H2A, and H2B after 4 hours, suggesting that activated macrophages modulate chromatin and release histones to regulate immune responses (136).

Macrophages clear NETs and histone debris to restore tissue homeostasis and prevent autoimmunity. This clearance involves DNase-I mediated degradation of NET DNA and C1q-assisted opsonization, via phagocytosis and macropinocytosis for lysosomal degradation (137). Pro-inflammatory macrophages exhibited increased macropinocytosis and active NET degradation, whereas uptake of apoptotic neutrophils skewed macrophages toward a pro-resolving phenotype (138). Notably, histone modifications within macrophages, particularly under metabolic dysregulation such as diabetes, predisposed them to a more inflammatory profile that impaired resolution pathways (139).

Overall, eHists orchestrate a self-amplifying thromboinflammatory circuit by activating monocytes and macrophages through TLR-dependent pathways, promoting cytokine release, tissue factor expression, NET propagation, and microvascular thrombosis, while endogenous counter-regulatory mechanisms such as CLU and macrophage-mediated clearance remain insufficient in severe disease.

### 5.5 Eicosanoids and pro-inflammatory cytokines in histone signaling

The mechanistic link between eHists and lipid mediators is established through the rapid surge of cytosolic calcium following receptor engagement. *Vulliamy* et al. demonstrated that histone H4, in particular, induces a sustained increase in cytosolic calcium in platelets, providing the surface area necessary for the assembly of procoagulant enzyme complexes. Pharmacologically, this calcium surge is a critical requirement for the activation of cytosolic phospholipase cPLA_2_, which facilitates the release of arachidonic acid (AA) from cell membrane phospholipids (140). The subsequent AA metabolism via the cyclooxygenase (COX) pathway generates thromboxane A^2^ (TXA^2^), a potent mediator that stabilizes thrombi alongside cytokines. Perez-Cremades et al. showed that eHists induce oxidative stress–dependent activation of NF-κB via TLR4, increasing COX/NOX expression in endothelial cells. This shift promotes a prothrombotic state, where TXA^2^ and proinflammatory prostaglandins enhance vascular permeability and recruit additional neutrophils and monocytes (87). A study in cervical cancer patients examined histone modification-associated genes (HMAGs) and their correlation with disease outcomes. Patients with low HMAG scores showed activation of arachidonic eicosanoid and unsaturated cell adhesion pathways, highlighting a link between histone modifications and eicosanoid-driven proinflammatory responses (141).

Apart from that, acetylation and deacetylation of histones by HAT and histone deacetylase (HDAC) respectively is closely related to the response of cells toward inflammation. In cases of oxidative stress that results in formation of reactive oxygen species (ROS), there is an induction in NF- κB pathway which further leads to pro-inflammatory effects by inducing HAT and inhibiting HDAC. Acetylation of histones allows DNA uncoiling, which makes the residues accessible to the transcription factors including NF- κB whereas deacetylation prevents its access to the transcription factors by creating tighter coils. Thus, involvement of histone acetylation status contributes to downstream effects of NF-κB mediated inflammation (142).

This synergistic interplay creates a robust feed-forward loop: lipid-derived eicosanoids provide rapid, localized effects like vasoconstriction and platelet activation, while protein-based cytokines sustain long-term leukocyte recruitment and tissue-level inflammation. Recognizing this integrated signaling landscape positions the dual inhibition of lipid and protein pathways as a sophisticated therapeutic target for mitigating the full spectrum of the thrombo-inflammatory response.

## 6 Immunogenicity based on histone subtypes

Specific histones exert different pathological effects, for example citrullinated H3 released by NETs promoted pro-inflammatory and pro-thrombotic responses, while H4 drove direct cytotoxicity and platelet activation (143). Furthermore, the functional landscape of chromatin is altered, and nucleosomes are given special characteristics when conventional histones are substituted by non-allelic isoforms of histones that have different structural characteristics (144).

### 6.1 Histone H1 (linker histone)

Histone H1 is a lysine-rich linker histone that organizes higher-order chromatin structure. Extracellularly, H1 is a component of NETs and can function as a DAMP. It has been reported to trigger inflammatory responses and may have a role in regulating apoptosis. In some contexts, H1 is found in the cytoplasm bound to lipid droplets and is released upon stimulation with endotoxin (145). Extracellular histone H1 possessed potent neurotoxic and pro-inflammatory properties though specific H1 variants demonstrated more nuanced intracellular roles (146). Among these, histone H1.2 has been identified as a key intracellular transducer of apoptosis following DNA damage. Konishi et al. demonstrated that upon genotoxic stress such as exposure to X-ray irradiation or etoposide, H1.2 is selectively released from the nucleus into the cytoplasm in a p53-dependent manner (147). Remarkably, of all H1 isoforms evaluated, only H1.2 triggered cytochrome c release from isolated mitochondria, a hallmark of intrinsic apoptosis, via a Bak-dependent pathway.

Neutrophil elastase (ELANE) and histone H1 isoforms synergistically eliminated cancer cells (148). ELANE, a serine protease released by human neutrophils during inflammation selectively killed various cancer cell types by proteolytically releasing the CD95 death domain, which interacted with histone H1 isoforms to trigger cell death pathways in cancer cells suggesting a promising selective and broad anticancer strategy (148). While H1 was identified as a NET component in some reports (27, 148–156), other studies suggested it was degraded during the process of NET formation (27, 148–156).

### 6.2 Histone H2A and H2A.Z (core histone and variant)

Histone H2A facilitates chromatin structure and exhibits unique immunogenic properties when released extracellularly. A notable variant, H2A.Z, has been implicated in inflammatory responses. Extracellular H2A contributed to tissue damage by interacting with cell membranes to induce pore formation and cell lysis (27, 148, 154–157). In sepsis patients, circulating H2A isoforms with vascular leakage and organ dysfunction (150). When released as a part of nucleosomes, H2A-H2B dimers during NET formation, activated TLR2 and amplified the inflammatory cascade (148, 151, 157). In heparin neutralization assays, H2A demonstrated procoagulant effects and acted synergistically with platelet factor 4 (PF4) to enhance clotting (149, 157). These findings highlight the multifaceted role of H2A in both inflammation and coagulation, making it a significant contributor to thromboinflammatory responses.

### 6.3 Histone H2B (core histone)

H2B serves as a significant Plasminogen receptor (PlgR) on leukocytes, including monocytes/macrophages, and participates in plasmin generation (149, 158–160). H2B bound to cell membranes via phosphatidylserine and negatively charged molecules like heparan sulfate (161). Increased surface H2B enhances plasminogen binding and plasmin generation, promoting inflammatory cell recruitment. Lysine analogues, such as ϵ-aminocaproic acid and Tranexamic acid (TXA) competed with plasminogen for H2B binding which inhibited macrophage migration (159).

### 6.4 Histone H3 (core histone; including citrullinated and acetylated forms)

Histone H3, particularly in its post-translationally modified forms, plays a pivotal role in immune activation and thrombogenesis. Citrullinated H3 (Cit-H3) exerts potent pro-inflammatory, pro-thrombotic effects, that drive chronic immune dysfunction (53, 161, 162). Cit-H3 interacted with TLR4, which led to NF-κB activation and subsequent cytokine release (53, 162–164). Elevated plasma levels of Cit-H3 were predictive of poor outcomes in sepsis and thrombotic conditions (164, 165). Acetylated H3 correlated with enhanced chromatin accessibility and increased transcription of pro-inflammatory genes during endothelial activation (166). Histone H3 bound directly to platelets via glycoprotein VI (GPVI), which induced platelet aggregation and granule release (102, 167, 168).

### 6.5 Histone H4 (core histone)

Among the five histone proteins, H4 exhibits the most pronounced immunogenic impact (168, 169). Histone H4 was identified through western blot analysis in the BAL fluid (BALF) samples of ARDS patients undergoing mechanical ventilation, whereas it was not detectable in BAL samples from individuals without health issues who served as healthy volunteers (169, 170). Upon membrane contact, H4 exhibited pore-forming abilities that resulted in lytic cell death (170, 171). H4 induced platelet ballooning via sustained increases in cytosolic calcium providing a surface area for assembly of procoagulant enzyme complexes and amplification of thrombin generation at sites of injury favoring a procoagulant transformation (140). Histone H4 p comprises a central histone-fold domain along with unstructured N-terminal and C-terminal tails and is said to be a physiological cofactor for prothrombin auto activation (171–174).

## 7 Pathways related to pro-thrombotic states

Histones enhanced thrombin generation in platelet-rich plasma (PRP) in a dose-dependent manner, even in the absence of external triggers (102). In purified systems, histones triggered platelet aggregation, P-selectin expression, phosphatidylserine exposure, FV/Va expression, and prothrombinase activity. Histone mediated activation of TLR2 and TLR4 stimulated the NF-κB pathway, promoting the expression of pro-inflammatory cytokines and adhesion molecules (174, 175). Furthermore, inhibiting platelet TLR2 and TLR4 with monoclonal antibodies decreased the proportion of activated platelets and reduced thrombin generation in PRP. Histones also induced the release of polyphosphate from platelets, which in turn activated the intrinsic pathway of the coagulation cascade independently of factor XII (FXII) (175, 176). *McDonald* et al., demonstrated that NET-bound-H4 interacted with platelets to trigger the release of inorganic polyphosphate from the platelets driving in intravascular coagulation *in vivo* (176, 177). This was the first study to report an *in vivo* infection model with dynamic NET-platelet-thrombin axis that promotes intravascular coagulation and microvascular dysfunction.

*Ammollo* et al., demonstrated that histones predominately H3 and H4 interfered with the activation of protein C (to APC) by thrombomodulin (TM), requiring the carboxyglutamic acid domain of protein C (124). Histones inhibited endothelial TM in a dose-dependent manner, thereby hampering the protein C-thrombomodulin anticoagulant system (178). APC displays antiapoptotic, cytoprotective as well as the anti-inflammatory properties (179). These effects occur via the cleavage of PAR-1 as part of a complex with endothelial cell protein C receptor (EPCR) via the activation of sphingosine 1-phosphate (S1P) and subsequent signaling cascades that enhance endothelial barrier function and reduce cellular apoptosis (124, 179, 180). TM also limits cytokine production in ECs and reduces the adhesion of leukocytes (such as E-selectin, ICAM-1, and VCAM-1) to these cells. Consequently, histones hinder the anti-inflammatory functions of the APC-thrombomodulin axis.

## 8 Clinical implications: histones as biomarkers

Extracellular histones and nucleosomes are active drivers of thromboinflammation, serving as prognostic indices and therapeutic targets. These circulating metrics of cytotoxic burden facilitate precise risk stratification. Notably, intact nucleosomes elicited potentiated immunogenicity relative to free histones, driven by the synergistic DAMP activity of the histone-DNA scaffold (181). In sepsis, elevated levels of histone H3 and CitH3) correlated strongly with disease severity, organ failure (e.g., AKI, DIC), inflammatory burden, and mortality, often aligning with clinical scoring systems like SOFA and APACHE II (181, 182). In adult and pediatric acute respiratory distress syndrome (ARDS and PARDS), elevated histone levels in plasma and bronchoalveolar lavage fluid are associated with increased organ dysfunction and poorer clinical outcomes (183–185). In ischemic stroke, elevated nucleosome levels, particularly on day 3 post-event- predicted long-term neurological recovery, while histone H3.1 served as a potential diagnostic and prognostic marker, correlated with inflammatory mediators such as IL-6 and VCAM-1 (182–184). In COVID-19-associated coagulopathy high circulating levels of histones, including CitH3, were linked to immunothrombosis, cardiac injury, and increased mortality, positioning them as potential early biomarkers for disease stratification and therapeutic targeting (183–185). Autoimmune disorders like systemic lupus erythematosus (SLE) and antiphospholipid syndrome (APS) also exhibit heightened extracellular histone signatures (184–187). In SLE, anti-histone and anti-nucleosome antibodies alongside modified nucleosomes like nitrated forms- are associated with disease activity and lupus nephritis. APS patients, while not routinely screened for circulating histones, display elevated NETs rich in histones, implicating them indirectly in thrombotic risk. While the diagnostic specificity of extracellular histones remains limited due to their ubiquity in inflammatory states, their utility as prognostic markers or as part of multiplex panels holds significant clinical promise.

## 9 Therapeutic strategies targeting extracellular histones

The pathogenic role of extracellular histones in thromboinflammation has spurred the development of diverse therapeutic strategies aimed at neutralizing their effects. While the systemic blockade of histones presents challenges due to their essential intracellular functions, targeting their extracellular, cytotoxic, and pro-thrombotic activities has spurred the development of several distinct pharmacological strategies (70, 186–188). These approaches include anti-histone antibodies, heparin and its derivatives, suramin, polyanions, and natural proteins such as C-reactive protein (CRP) and osteopontin as summarized in **Table 2**. Additional strategies target histone release or modification, including PAD inhibitors, HDAC inhibitors, α^2^-adrenoceptor agonists, and agents that promote histone degradation, such as Activated Protein C (APC), recombinant thrombomodulin (rTM), and proteases like Factor VII-activating protease (FSAP) (**Table 2**) (71, 192, 200, 210, 211, 215–219).

**Table 2:** Current and emerging therapeutic strategies targeting extracellular histones in thromboinflammation: This summary categorizes pharmacological interventions by their primary mechanism of action: direct neutralization, inhibition of release, enzymatic degradation or physical removal.

Therapeutic strategy	Mechanism of action	Key preclinical findings	Key clinical findings/trial status	Potential advantages	Potential disadvantages/ limitations	References
I. Direct neutralization & sequestration						
**Anti-histone antibodies**	Neutralize extracellular histones	Reduced toxicity, inflammation, organ injury; improved survival in sepsis, trauma, lung injury models	Preclinical; some inconsistency in efficacy reported	Specific targeting of histones	Development of reliable and effective antibodies; delivery; cost	(189, 190)
**Heparin and derivates**	Bind and neutralize histones via electrostatic interactions; reduce histone release	Reduced inflammatory markers, organ injury (lung, kidney, intestine); improved survival in sepsis/ARDS models	Widely used anticoagulant; non-anticoagulant versions in development	Readily available; known pharmacology	Bleeding risk with anticoagulant doses; optimal dosing for histone neutralization unclear	(191–193)
**Small polyaniions** (eg.Suramin, STC3141)	Polyanionic drug, binds and neutralizes histones	Reduced histone-induced thrombin generation, EC protection, prevented lung injury & mortality in mice	Preclinical	Broad histone binding	Less affinity for citrullinated histones; potential side effects of suramin	(194, 195)
**Recombinant thrombomodulin (rTM)**	Lectin-like domain binds and sequesters extracellular histones; supports APC generation.	Protection against histone-induced endothelial dysfunction and death in sepsis models.	Clinical. Approved in Japan/select regions for DIC; efficacy in histone pathology supported by trial data.	Dual mechanism: neutralizes histones directly and supports anticoagulant pathways.	Availability varies by region (not universally approved for sepsis).	(196, 197)
**Endogenous scavengers** (CRP, Albumin)	Physiologic buffering of circulating histones to prevent cell membrane damage.	CRP and Albumin effectively buffer histone toxicity *in vitro* and in limited *in vivo* models.	N/A. These are endogenous proteins; therapeutic supplementation is investigational.	Utilizes natural host defense mechanisms; low immunogenicity.	High physiological concentrations required for therapeutic effect; albumin is non-specific.	(198–200)
II. Inhibition of histone release						
**PAD inhibitors** (e.g., GSK484, Cl-amidine)	Inhibit PAD4, reduce histone citrullination and NETosis, decrease histone release	Reduced NETosis, histone release, organ damage in various models	Preclinical; some clinical trials for other inflammatory diseases	Targets a key modification step	Specificity; potential impact on physiological NETosis	(201–203)
**HDAC inhibitors**	Modulate histone acetylation; complex effects on NETosis	Low doses may promote NETosis; higher doses suppress NETosis, shift to apoptosis.	Preclinical for NET modulation; some HDACi in cancer/inflammatory disease trials	Modulates histone release/PTMs	Dose-dependent opposing effects; specificity	(204, 205)
III. Enzymatic degradation						
**Activated protein C (APC)**	APC degrades histones by proteolytically cleaving them.	Reduced organ injury, improved survival in sepsis/histone-infusion models	Beeding risk limited use. APC variants in development	Targets histone directly (APC)	Bleeding risk.	(206, 207)
**Factor VII-activating protease (FSAP)**	Proteolytically cleaves extracellular histones (specifically H3 and H4), neutralizing their cytotoxicity	FSAP efficiently degrades histones in plasma; FSAP-deficient plasma shows impaired histone neutralization and increased cytotoxicity.	Preclinical / Genetic Association. Humans with the "Marburg I" polymorphism have higher risk of thrombosis and liver fibrosis (20).	Functions endogenously without needing drug administration (in healthy individuals)	Genetic variability: ~5% of the population carries the Marburg I variant (inactive FSAP), making them more vulnerable to histone injury.	(208–211)
IV. Physical removal						
**Extracorporeal removal** (eg., Cytosorb, AN69ST membrane)	Physical adsorption of histones from blood	Effective removal in vitro and in some ex vivo/animal models	Investigational; devices like CytoSorb used in sepsis for cytokine removal	Rapid removal of high histone loads	Non-specific; impact on other blood components; feasibility	(212–214)

The status of each strategy ranges from preclinical investigation to clinically approved agents repurposed for histone suppression. **APC**, Activated Protein C; **CRP**, C-reactive protein; **NAH**, non-anticoagulant heparin; **NETs**, neutrophil extracellular traps; **PAD**, peptidylarginine deiminase; **rTM**, recombinant thrombomodulin.

The therapeutic landscape for mitigating thromboinflammation increasingly prioritizes the neutralization of eHists and NETs to protect endothelial integrity and actively promote thrombus resolution (70, 187, 188). Small polyanionic molecules have emerged as primary candidates in clinical development due to their ability to electrostatically neutralize circulating histones without the severe hemorrhagic risks associated with systemic anticoagulation (17). Specifically, β-O-methyl cellobiose sulfate (STC3141) has demonstrated significant clinical translation, currently progressing through Phase II trials for conditions characterized by systemic hyperinflammation and coagulopathy, such as sepsis and acute respiratory distress syndrome (194, 218–220). Concurrently, preclinical investigations into the repurposed polyanion suramin have elucidated its efficacy in forming stable electrostatic complexes with individual histones (194, 195, 220). This neutralization preserves the vascular endothelium by actively attenuating histone-mediated thrombin generation and preventing subsequent capillary hyperpermeability.

In scenarios of profound hyperinflammatory shock where pharmacological intervention alone is insufficient, extracorporeal blood purification strategies are increasingly utilized in the intensive care setting (195, 220, 221). Devices employing heparin-functionalized adsorbents, such as the CytoSorb cartridge, facilitate the physical clearance of positively charged DAMPs including eHists and HMGB1, directly from the systemic circulation (195, 221–224). This physical extraction provides substantial mechanistic protection to the endothelial barrier, significantly mitigating capillary leak syndrome and reducing the total volume of fluid resuscitation required in critically ill septic cohorts (193, 221–225). Alternatively, the upstream inhibition of NETosis via PAD4 inhibitors, such as GSK484, presents a targeted mechanism to prevent histone citrullination and subsequent chromatin release prior to endothelial exposure (193, 225–227). However, recent *in vivo* models suggest that while pharmacological PAD4 inhibition effectively reduces localized NET density, it may not independently ameliorate broader systemic inflammatory biomarkers or restore tissue architecture (193, 225–227). These findings suggest that successfully targeting eHists to drive proper thrombus resolution may necessitate combinatorial therapeutic approaches, pairing PAD4 inhibition with direct histone neutralizers, rather than relying on isolated upstream inhibition alone.

## 10 Conclusions and future directions

Extracellular histones have emerged as pivotal mediators at the intersection of inflammation and thrombosis, transforming from structural nuclear proteins into potent damage-associated molecular patterns (DAMPs) that drive thrombo-inflammatory cascades. Their ability to disrupt cellular membranes, activate pattern recognition receptors such as TLR2, TLR4, and TLR9, and amplify immune responses underscores their central role in endothelial dysfunction, platelet activation, and neutrophil-driven NETosis. Post-translational modifications (PTMs), including citrullination, acetylation, methylation, and lactylation, further diversifying histone functions, influencing their immunogenicity, cytotoxicity, and procoagulant potential. These mechanistic insights position extracellular histones not merely as biomarkers but as causal agents in disease progression across sepsis, ARDS, stroke, COVID-19, and autoimmune disorders.

Despite significant advances, critical gaps remain. The structural context of histone release (free histones versus nucleosome) requires deeper exploration to delineate receptor specificity and signaling outcomes. Similarly, the interplay between histone PTMs and metabolic states, and their impact on thrombo-inflammatory severity, warrant systematic investigation. Another unresolved question is the differential pathogenicity of histone subtypes and variants, which may hold the key to precision-targeted therapies.

Future research should prioritize mechanistic studies to map histone-receptor interactions and downstream signaling, including crosstalk with inflammasomes and RAGE pathways. It should also focus on PTM-driven investigations to understand how modifications modulate extracellular histone activity and therapeutic susceptibility. Development of targeted interventions, such as PTM-specific inhibitors, neutralizing antibodies, and membrane-stabilizing agents, with emphasis on minimizing off-target effects, is essential. Large-scale clinical studies are needed to validate histone signatures as biomarkers for disease stratification and prognosis. Innovative therapeutic strategies, including extracorporeal removal systems and combination approaches that integrate histone neutralization with anti-inflammatory and anticoagulant modalities, should be explored.

By addressing these gaps, future work can translate the growing mechanistic understanding of histone biology into clinically actionable strategies, ultimately mitigating the burden of thrombo-inflammatory diseases.
